# 2-(4-Amino­pyridinio)acetate

**DOI:** 10.1107/S1600536809009258

**Published:** 2009-03-25

**Authors:** Ge Liu

**Affiliations:** a Chifeng University, Chifeng 024000, People’s Republic of China

## Abstract

In the title compound, C_7_H_8_N_2_O_2_, the dihedral angle between the pyridinium ring and the carboxyl­atomethyl group is 74.5 (1)°. Strong inter­molecular N—H⋯O hydrogen bonds between the amine and carboxyl­ate groups form a layered hydrogen-bonded network perpendicular to [010]. In addition, there are some weak C—H⋯O hydrogen bonds present in the structure.

## Related literature

For the biological activity of pyridinium derivatives, see: Sliwa & Mianowska (1989 ▶). For hydrogen-bond definitions, see: Desiraju & Steiner (1999 ▶). For the analysis of bond order, see: Ludvík *et al.* (2007 ▶). For the Cambridge Structural Database (Version 5.30 and addenda up to 12th February 2009), see: Allen (2002 ▶). 
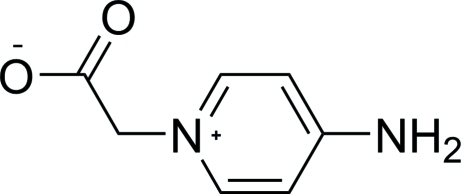


## Experimental

### 

#### Crystal data

C_7_H_8_N_2_O_2_
*M* *_r_* = 152.15
Monoclinic, 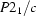

*a* = 8.9766 (18) Å
*b* = 9.0555 (18) Å
*c* = 8.9886 (18) Å
β = 106.57 (3)°
*V* = 700.3 (2) Å^3^
*Z* = 4
Mo *K*α radiation
μ = 0.11 mm^−1^
*T* = 293 K
0.16 × 0.14 × 0.12 mm

#### Data collection

Rigaku R-AXIS RAPID-S diffractometer
Absorption correction: none
7228 measured reflections
1599 independent reflections
1123 reflections with *I* > 2σ(*I*)
*R*_int_ = 0.052

#### Refinement

*R*[*F* ^2^ > 2σ(*F* ^2^)] = 0.055
*wR*(*F* ^2^) = 0.117
*S* = 1.11
1599 reflections
106 parameters
2 restraints
H atoms treated by a mixture of independent and constrained refinement
Δρ_max_ = 0.17 e Å^−3^
Δρ_min_ = −0.20 e Å^−3^

### 

Data collection: *RAPID-AUTO* (Rigaku, 1998 ▶); cell refinement: *RAPID-AUTO*; data reduction: *CrystalStructure* (Rigaku/MSC, 2002 ▶); program(s) used to solve structure: *SHELXS97* (Sheldrick, 2008 ▶); program(s) used to refine structure: *SHELXL97* (Sheldrick, 2008 ▶); molecular graphics: *SHELXTL* (Sheldrick, 2008 ▶) and *Mercury* (Macrae *et al.*, 2006 ▶); software used to prepare material for publication: *SHELXTL*.

## Supplementary Material

Crystal structure: contains datablocks I, global. DOI: 10.1107/S1600536809009258/fb2140sup1.cif

Structure factors: contains datablocks I. DOI: 10.1107/S1600536809009258/fb2140Isup2.hkl

Additional supplementary materials:  crystallographic information; 3D view; checkCIF report

## Figures and Tables

**Table 1:** Hydrogen-bond geometry (Å, °)

*D*—H⋯*A*	*D*—H	H⋯*A*	*D*⋯*A*	*D*—H⋯*A*
N1—H1*A*⋯O2^i^	0.859 (16)	2.095 (16)	2.946 (2)	170 (2)
N1—H1*A*⋯O1^i^	0.859 (16)	2.605 (19)	3.265 (2)	134.5 (18)
N1—H1*B*⋯O2^ii^	0.881 (15)	2.041 (17)	2.891 (2)	162 (2)
C3—H3⋯O1^iii^	0.93	2.42	3.334 (3)	166
C4—H4⋯O1^iv^	0.93	2.38	3.247 (3)	155
C6—H6*A*⋯O1^iv^	0.97	2.49	3.359 (3)	149

Symmetry codes: (i) 
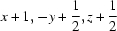
; (ii) 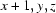; (iii) 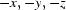; (iv) 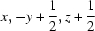.

**Fig. 1.**
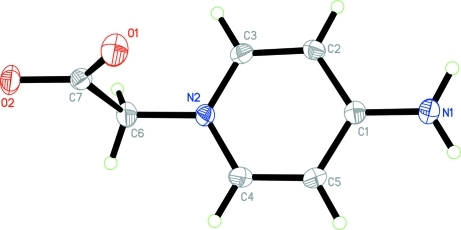
The structure of 4-amino-pyridinium-N-acetate with the displacement ellipsoids shown at the 30% probability level.

**Fig. 2.**
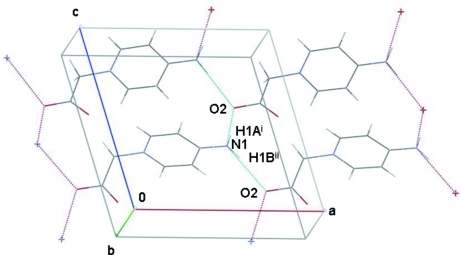
A depiction of the layer perpendicular to b axis with the N1—H1A···O2 and N1—H1B···O2 hydrogen bonds. Symmetry codes: (i) x + 1, -y + 1/2, z + 1/2; (ii) x + 1, y, z.

## supplementary crystallographic information

### Comment

It is known that pyridinium derivatives have antibacterial and fungicidal activities (Sliwa & Mianowska, 1989). As 4-amino-pyridinium-*N*-acetate contains both amino and carboxylate groups, it may form interesting hydrogen-bonded network. Therefore the crystal structure analysis of the title compound has been undertaken. The molecular structure of the title structure is shown in Fig. 1. The dihedral angle between the planes of pyridinium ring and carboxymethylene fragment is 74.5°. Strong (Desiraju & Steiner, 1999) intermolecular N1—H1A···O2^i^ and N1—H1B···O2^ii^ (i: x + 1, -y + 1/2, z + 1/2; ii: x + 1, y, z) hydrogen bonds form the zig-zagged layer perpendicular to [010] (Fig. 2; Table 1), O2 is the acceptor of both amine hydrogens. In addition, weak hydrogen bonds between the pyridinium ring, methylene and the carboxylate groups, *i.e.* C4—H4···O1^iv^ and C6—H6A···O1^iv^ (iv: x, -y + 1/2, z + 1/2), are also involved in these layers. C3—H3···O1^iii^ (iii: -x, -y, -z) hydrogen bonds between the pyridinium ring and the carboxylate groups interconnect the neighbouring layers (Table 1).

### Experimental

A solution of 4-aminopyridine (5.46 g, 0.058 mol), 1-chloroacetic acid (13.1 g, 0.139 mol) and Na_2_CO_3_ (16.6 g, 0.157 mol) in 110 ml of H_2_O was stirred for 3 h at 373 K. Then the solution was acidified by concentrated HCl to pH = 2. The solution was left overnight in a refrigerator, the precipitation was filtered, affording colourless block shaped (about 0.12 mm - 0.14 mm) crystals of 4-amino-pyridinium-*N*-acetate.

### Refinement

All the hydrogens were discernible in the difference electron density map. All the H atoms except the amine group were placed into the geometrically idealized positions and constrained to ride on their parent atoms with C_methylene_—H = 0.97 Å, C_aryl_—H = 0.93 Å. *U*_iso_(H) = 1.2*U*_eq_(C_parent_). The distances N—H of the amine hydrogens were restrained to 0.86 (2) Å because this group is involved in the hydrogen bond pattern, the bond order of C1—N1 (1.331 (2) Å) is about 1.5 (Ludvík *et al.*,  2007) and the result of the search in the Cambridge Crystallographic Structure Database (Allen, 2002; Version 5.30 and addenda up to 12th February 2009) gave the N_amine_—H···O about 160° as the most probable result. The displacement parameters of the amine hydrogens were constrained: *U*_iso_(H) = 1.2*U*_eq_(N).

### Crystal data

**Table:**

C_7_H_8_N_2_O_2_	*F*(000) = 320
*M**_r_* = 152.15	*D*_x_ = 1.443 Mg m^−^^3^
Monoclinic, *P*2_1_/*c*	Mo *K*α radiation, λ = 0.71073 Å
Hall symbol: -P 2ybc	Cell parameters from 5894 reflections
*a* = 8.9766 (18) Å	θ = 3.3–27.6°
*b* = 9.0555 (18) Å	µ = 0.11 mm^−^^1^
*c* = 8.9886 (18) Å	*T* = 293 K
β = 106.57 (3)°	Prism, colourless
*V* = 700.3 (2) Å^3^	0.16 × 0.14 × 0.12 mm
*Z* = 4	

### Data collection

**Table:**

Rigaku R-AXIS RAPID-S diffractometer	1123 reflections with *I* > 2σ(*I*)
Radiation source: fine-focus sealed tube	*R*_int_ = 0.052
graphite	θ_max_ = 27.5°, θ_min_ = 3.3°
ω scans	*h* = −11→11
7228 measured reflections	*k* = −11→11
1599 independent reflections	*l* = −11→11

### Refinement

**Table:**

Refinement on *F*^2^	Primary atom site location: structure-invariant direct methods
Least-squares matrix: full	Secondary atom site location: difference Fourier map
*R*[*F*^2^ > 2σ(*F*^2^)] = 0.055	Hydrogen site location: difference Fourier map
*wR*(*F*^2^) = 0.117	H atoms treated by a mixture of independent and constrained refinement
*S* = 1.11	*w* = 1/[σ^2^(*F*_o_^2^) + (0.0391*P*)^2^ + 0.2309*P*] where *P* = (*F*_o_^2^ + 2*F*_c_^2^)/3
1599 reflections	(Δ/σ)_max_ < 0.001
106 parameters	Δρ_max_ = 0.17 e Å^−^^3^
2 restraints	Δρ_min_ = −0.20 e Å^−^^3^
26 constraints	

### Special details

**Table:**

Geometry. All e.s.d.'s (except the e.s.d. in the dihedral angle between two l.s. planes) are estimated using the full covariance matrix. The cell e.s.d.'s are taken into account individually in the estimation of e.s.d.'s in distances, angles and torsion angles; correlations between e.s.d.'s in cell parameters are only used when they are defined by crystal symmetry. An approximate (isotropic) treatment of cell e.s.d.'s is used for estimating e.s.d.'s involving l.s. planes.
Refinement. Refinement of *F*^2^ against ALL reflections. The weighted *R*-factor *wR* and goodness of fit *S* are based on *F*^2^, conventional *R*-factors *R* are based on *F*, with *F* set to zero for negative *F*^2^. The threshold expression of *F*^2^ > σ(*F*^2^) is used only for calculating *R*-factors(gt) *etc*. and is not relevant to the choice of reflections for refinement. *R*-factors based on *F*^2^ are statistically about twice as large as those based on *F*, and *R*- factors based on ALL data will be even larger.

### Fractional atomic coordinates and isotropic or equivalent isotropic displacement parameters (Å^2^)

**Table:**

	*x*	*y*	*z*	*U*_iso_*/*U*_eq_	
C1	0.4649 (2)	0.1375 (2)	0.3639 (2)	0.0269 (4)	
C2	0.3811 (2)	0.0336 (2)	0.2546 (2)	0.0281 (5)	
H2	0.4307	−0.0174	0.1924	0.034*	
C3	0.2291 (2)	0.0078 (2)	0.2398 (2)	0.0296 (5)	
H3	0.1759	−0.0610	0.1672	0.035*	
C4	0.2291 (2)	0.1803 (2)	0.4331 (2)	0.0338 (5)	
H4	0.1760	0.2300	0.4929	0.041*	
C5	0.3814 (2)	0.2103 (2)	0.4532 (2)	0.0346 (5)	
H5	0.4313	0.2797	0.5268	0.042*	
C6	−0.0153 (2)	0.0564 (2)	0.2986 (2)	0.0321 (5)	
H6A	−0.0465	0.0953	0.3858	0.039*	
H6B	−0.0364	−0.0488	0.2927	0.039*	
C7	−0.1133 (2)	0.1297 (2)	0.1497 (2)	0.0269 (4)	
N1	0.6143 (2)	0.1661 (2)	0.3810 (2)	0.0361 (5)	
H1A	0.662 (2)	0.228 (2)	0.450 (2)	0.043*	
H1B	0.658 (2)	0.131 (2)	0.312 (2)	0.043*	
N2	0.15247 (17)	0.07944 (18)	0.32765 (17)	0.0273 (4)	
O1	−0.04740 (16)	0.19659 (17)	0.06662 (16)	0.0407 (4)	
O2	−0.25727 (15)	0.11367 (17)	0.12513 (16)	0.0393 (4)	

### Atomic displacement parameters (Å^2^)

**Table:**

	*U*^11^	*U*^22^	*U*^33^	*U*^12^	*U*^13^	*U*^23^
C1	0.0265 (10)	0.0294 (10)	0.0235 (10)	0.0018 (8)	0.0052 (8)	0.0062 (8)
C2	0.0303 (11)	0.0285 (11)	0.0263 (10)	0.0032 (8)	0.0093 (8)	−0.0020 (8)
C3	0.0312 (11)	0.0311 (11)	0.0243 (10)	−0.0003 (9)	0.0045 (8)	−0.0023 (9)
C4	0.0335 (11)	0.0383 (12)	0.0298 (11)	0.0031 (9)	0.0093 (9)	−0.0080 (10)
C5	0.0327 (11)	0.0377 (12)	0.0320 (11)	−0.0034 (10)	0.0070 (9)	−0.0123 (10)
C6	0.0258 (11)	0.0387 (12)	0.0316 (11)	0.0001 (9)	0.0078 (8)	0.0063 (10)
C7	0.0268 (11)	0.0277 (10)	0.0250 (10)	0.0013 (8)	0.0053 (8)	−0.0013 (8)
N1	0.0273 (10)	0.0454 (12)	0.0349 (11)	−0.0065 (8)	0.0079 (8)	−0.0043 (9)
N2	0.0216 (8)	0.0353 (10)	0.0233 (8)	0.0026 (7)	0.0035 (6)	0.0012 (7)
O1	0.0358 (8)	0.0505 (10)	0.0352 (8)	−0.0031 (7)	0.0089 (7)	0.0149 (7)
O2	0.0214 (8)	0.0584 (10)	0.0354 (8)	0.0015 (7)	0.0038 (6)	0.0053 (7)

### Geometric parameters (Å, °)

**Table:**

C1—N1	1.331 (2)	C5—H5	0.9300
C1—C5	1.410 (3)	C6—N2	1.468 (2)
C1—C2	1.412 (3)	C6—C7	1.528 (3)
C2—C3	1.353 (3)	C6—H6A	0.9700
C2—H2	0.9300	C6—H6B	0.9700
C3—N2	1.351 (2)	C7—O1	1.236 (2)
C3—H3	0.9300	C7—O2	1.256 (2)
C4—C5	1.354 (3)	N1—H1A	0.859 (16)
C4—N2	1.354 (3)	N1—H1B	0.881 (15)
C4—H4	0.9300		
			
N1—C1—C5	121.68 (19)	N2—C6—C7	113.54 (16)
N1—C1—C2	122.00 (18)	N2—C6—H6A	108.9
C5—C1—C2	116.32 (17)	C7—C6—H6A	108.9
C3—C2—C1	120.36 (18)	N2—C6—H6B	108.9
C3—C2—H2	119.8	C7—C6—H6B	108.9
C1—C2—H2	119.8	H6A—C6—H6B	107.7
N2—C3—C2	121.80 (18)	O1—C7—O2	126.61 (18)
N2—C3—H3	119.1	O1—C7—C6	119.14 (17)
C2—C3—H3	119.1	O2—C7—C6	114.24 (17)
C5—C4—N2	121.39 (19)	C1—N1—H1A	119.6 (15)
C5—C4—H4	119.3	C1—N1—H1B	118.9 (15)
N2—C4—H4	119.3	H1A—N1—H1B	121 (2)
C4—C5—C1	120.72 (19)	C3—N2—C4	119.40 (17)
C4—C5—H5	119.6	C3—N2—C6	119.69 (17)
C1—C5—H5	119.6	C4—N2—C6	120.71 (16)
			
N1—C1—C2—C3	−179.64 (18)	N2—C6—C7—O2	−178.50 (17)
C5—C1—C2—C3	−0.2 (3)	C2—C3—N2—C4	0.3 (3)
C1—C2—C3—N2	0.0 (3)	C2—C3—N2—C6	175.09 (17)
N2—C4—C5—C1	0.3 (3)	C5—C4—N2—C3	−0.5 (3)
N1—C1—C5—C4	179.46 (19)	C5—C4—N2—C6	−175.22 (18)
C2—C1—C5—C4	0.0 (3)	C7—C6—N2—C3	−72.5 (2)
N2—C6—C7—O1	1.6 (3)	C7—C6—N2—C4	102.2 (2)

### Hydrogen-bond geometry (Å, °)

**Table:**

*D*—H···*A*	*D*—H	H···*A*	*D*···*A*	*D*—H···*A*
N1—H1A···O2^i^	0.86 (2)	2.10 (2)	2.946 (2)	170 (2)
N1—H1A···O1^i^	0.86 (2)	2.61 (2)	3.265 (2)	135 (2)
N1—H1B···O2^ii^	0.88 (2)	2.04 (2)	2.891 (2)	162 (2)
C3—H3···O1^iii^	0.93	2.42	3.334 (3)	166
C4—H4···O1^iv^	0.93	2.38	3.247 (3)	155
C6—H6A···O1^iv^	0.97	2.49	3.359 (3)	149

Symmetry codes: (i) *x*+1, −*y*+1/2, *z*+1/2; (ii) *x*+1, *y*, *z*; (iii) −*x*, −*y*, −*z*; (iv) *x*, −*y*+1/2, *z*+1/2.
